# Prospective identification of resistance mechanisms to HSP90 inhibition in KRAS mutant cancer cells

**DOI:** 10.18632/oncotarget.13841

**Published:** 2016-12-09

**Authors:** Arefeh Rouhi, Christina Miller, Sarah Grasedieck, Stefanie Reinhart, Britta Stolze, Hartmut Döhner, Florian Kuchenbauer, Lars Bullinger, Stefan Fröhling, Claudia Scholl

**Affiliations:** ^1^ Department of Internal Medicine III, Ulm University, Ulm, Germany; ^2^ Department of Translational Oncology, National Center for Tumor Diseases (NCT) Heidelberg and German Cancer Research Center (DKFZ), Heidelberg, Germany; ^3^ Section for Personalized Oncology, Heidelberg University Hospital, Heidelberg, Germany; ^4^ German Cancer Consortium (DKTK), Heidelberg, Germany

**Keywords:** drug resistance, HSP90 inhibition, PU-H71, mutant KRAS, MDR1

## Abstract

Inhibition of the HSP90 chaperone results in depletion of many signaling proteins that drive tumorigenesis, such as downstream effectors of KRAS, the most commonly mutated human oncogene. As a consequence, several small-molecule HSP90 inhibitors are being evaluated in clinical trials as anticancer agents. To prospectively identify mechanisms through which HSP90-dependent cancer cells evade pharmacologic HSP90 blockade, we generated multiple mutant KRAS-driven cancer cell lines with acquired resistance to the purine-scaffold HSP90 inhibitor PU-H71. All cell lines retained dependence on HSP90 function, as evidenced by sensitivity to short hairpin RNA-mediated suppression of HSP90AA1 or HSP90AB1 (also called HSP90α and HSP90β, respectively), and exhibited two types of genomic alterations that interfere with the effects of PU-H71 on cell viability and proliferation: (i) a Y142N missense mutation in the ATP-binding domain of HSP90α that co-occurred with amplification of the HSP90AA1 locus, (ii) genomic amplification and overexpression of the ABCB1 gene encoding the MDR1 drug efflux pump. In support of a functional role for these alterations, exogenous expression of HSP90α Y142N conferred PU-H71 resistance to HSP90-dependent cells, and pharmacologic MDR1 inhibition with tariquidar or lowering ABCB1 expression restored sensitivity to PU-H71 in ABCB1-amplified cells. Finally, comparison with structurally distinct HSP90 inhibitors currently in clinical development revealed that PU-H71 resistance could be overcome, in part, by ganetespib (also known as STA9090) but not tanespimycin (also known as 17-AAG). Together, these data identify potential mechanisms of acquired resistance to small molecules targeting HSP90 that may warrant proactive screening for additional HSP90 inhibitors or rational combination therapies.

## INTRODUCTION

Heat shock protein 90 (HSP90) constitutes a functionally diverse superfamily of highly conserved chaperone proteins that aid in the proper folding, assembly and localization of many cellular proteins, termed “clients”, as well as protecting proteins from proteasomal degradation. The two chaperones HSP90α (encoded by *HSP90AA1*) and HSP90β (encoded by *HSP90AB1*) are localized in the cytoplasm and comprise a large fraction of cytosolic proteins in unstressed cells [1, 2]. Among the more than 200 HSP90 clients are many proteins required for tumor growth, including primary cancer “drivers” such as BCR-ABL1, wildtype ERBB2 and mutant forms of EGFR, ERBB2, FLT3, JAK2 and KIT, as well as critical downstream effectors of these oncoproteins such as AKT1 and RAF1 [3].

The capacity of HSP90 to protect key oncogenic proteins from degradation has spurred the development of small molecules that interfere with the conformational cycling of HSP90 through binding to its N-terminal nucleotide pocket and locking it in a nucleotide-bound form [4], which in turn leads to the depletion of misfolded, inactive or unstable client proteins. There are, or have been, nearly 20 HSP90 inhibitors in clinical development [5], which are structurally classified as derivatives of geldanamycin, resorcinol, purine, dihydroindazolone, dihydropyridopyrimidine and tropane [6]. For example, the geldanamycin derivative tanespimycin (also called 17-AAG) was the first HSP90 inhibitor that entered clinical trials [7], and PU-H71 is a newer and more efficient, water-soluble purine analog [8] that is currently being tested in two phase 1 trials (ClinicalTrials.gov Identifiers NCT01581541 and NCT01393509). The most promising clinical results with these drugs have been achieved in cancers that are addicted to certain HSP90 clients. For example, a phase 2 trial demonstrated that the combined administration of trastuzumab and tanespimycin in patients with trastuzumab-resistant ERBB2-positive breast cancer was associated with a clinical benefit rate of 59% and a median progression-free and overall survival of six and 17 months, respectively [9]. Another phase 2 study showed that the resorcinol-derived HSP90 inhibitor ganetespib (also called STA9090) has clinical activity in trastuzumab-resistant ERBB2-positive as well as triple-negative breast cancer [10].

Preclinical data suggest that HSP90 inhibition could also be effective in cancers that acquired resistance to other targeted therapeutics, such as mutant EGFR-driven lung adenocarcinoma [11]. Common mechanisms of resistance to ATP-competitive EGFR tyrosine kinase inhibitors such as gefitinib and erlotinib are the T790M “gatekeeper” mutation in the EGFR kinase domain [12], and amplification and/or overexpression of the MET and AXL receptor tyrosine kinases [13, 14], which take over the function of inhibited EGFR to sustain tumor growth. This so-called “oncogenic switch” may be overcome by HSP90 inhibition since EGFR, MET, AXL and BRAF are all HSP90 clients [15]. In line with this, Xu et al. (2012) could show that combined EGFR and HSP90 inhibitor treatment efficiently reduced the growth of lung tumors driven by EGFR^T790M^ and MET in mice [11].

Due to their pleiotropic effects on multiple signaling proteins, HSP90 inhibitors may also prove useful for the treatment of cancers driven by oncoproteins that are difficult to target directly. For example, among the diverse substrates of HSP90 are several immediate downstream effectors as well as more “distant” candidate synthetic lethal interactors of mutant *KRAS*, the most common human oncogene which has evaded all attempts at therapeutic targeting so far [16–20]. Indeed, molecularly stratified clinical trials of HSP90 inhibitors include, for example, patients with KRAS mutant lung cancer (ClinicalTrials.gov Identifier NCT01124864).

Previous studies have extensively investigated the near-obligatory development of resistance in cancers treated with kinase inhibitors [21]. In contrast, it is largely unknown whether and how cancer cells can also escape the effects of HSP90 blockade; however, the preclinical observation that cultured glioblastoma cells developed tanespimycin resistance due to reduced expression of the NAD(P)H dehydrogenase quinone 1, a cellular enzyme responsible for modifying tanespimycin for increased binding to HSP90 [22], indicates that clinical resistance will occur as more patients are being treated with these agents.

The discovery of several clinically relevant resistance mechanisms to targeted cancer drugs, for example mutations affecting FLT3^N676^ or FLT3^G697^ in acute myeloid leukemia [23–25], amplification of the *MET* gene in EGFR mutant lung adenocarcinoma [13] or BRAF^V600E^ splice variants in melanoma [26], was achieved through systematic analysis of *in vitro* cancer models with experimentally induced insensitivity to the respective agents. Here, we have established multiple mutant KRAS-driven cancer cell lines with acquired resistance to the purine-scaffold HSP90 inhibitor PU-H71 to prospectively identify mechanisms through which HSP90-dependent cancer cells evade pharmacologic HSP90 blockade.

## RESULTS

### Generation of PU-H71-resistant cancer cell lines

**Figure 1:**
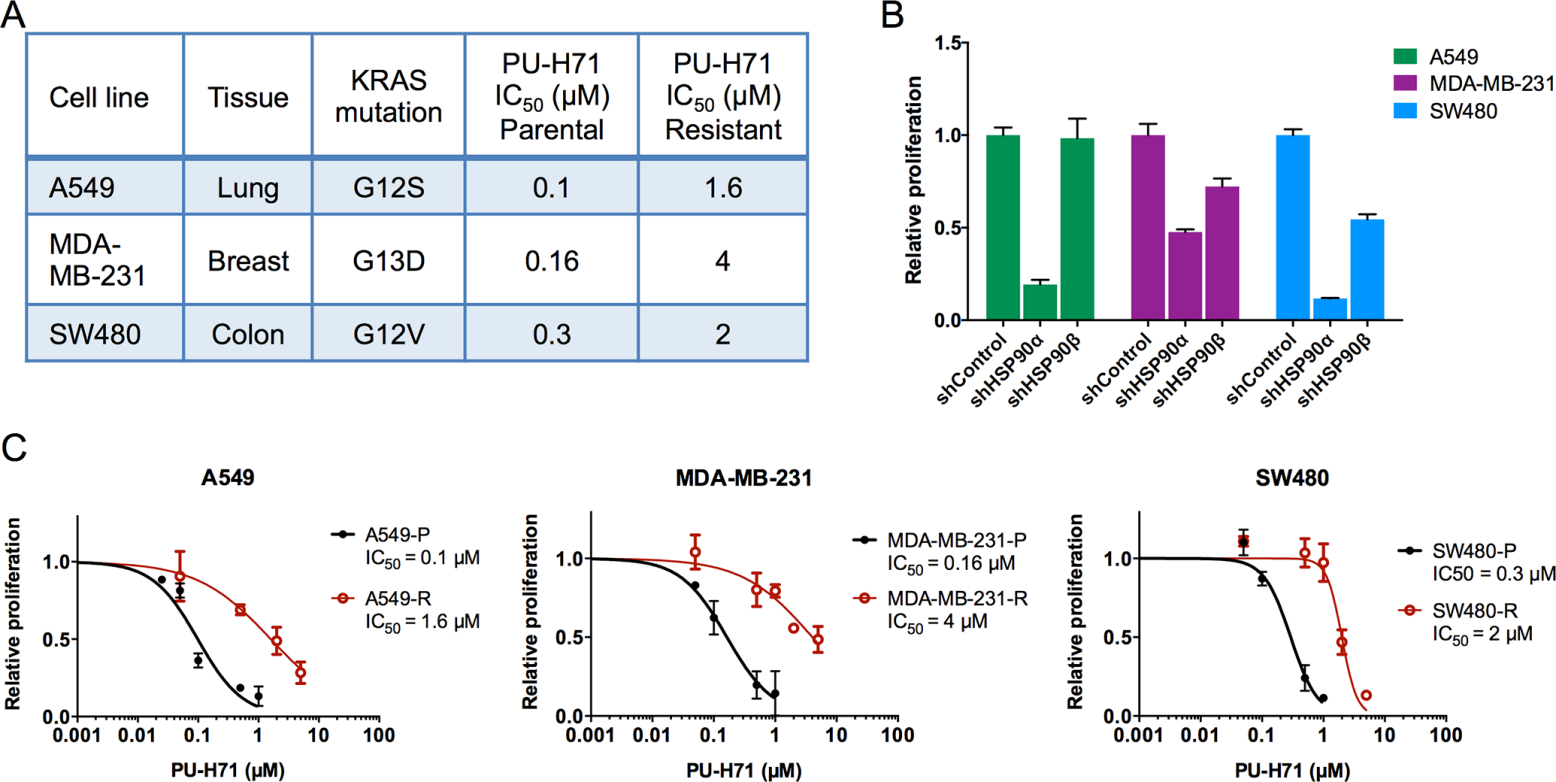
Generation of PU-H71-resistant cancer cell lines (**A**) characteristics of the cell lines used for subsequent analyses. (**B**) viability and proliferation of parental cell lines seven days after transduction with shRNAs targeting HSP90α or HSP90β, or a non-targeting control shRNA. (**C**) dose-response curves and IC_50_ values for PU-H71-sensitive parental and PU-H71-resistant cell lines. Proliferation was measured four days post drug treatment and normalized to untreated controls. Experiments were performed in triplicate, and one of two independent experiments is shown. Data are represented as mean ± SEM.

To identify mechanisms of resistance to HSP90 inhibition, we chose three KRAS mutant cell lines (A549, MDA-MB-231, SW480) that are derived from different cancer types (lung adenocarcinoma, triple-negative breast cancer, colorectal cancer) and exhibit dependence on the expression of mutant KRAS and the HSP90 client protein STK33 [16, 18] (Figure 1A). Short hairpin RNA-mediated knockdown experiments demonstrated that all three cell lines required HSP90α for their viability and proliferation, and that MDA-MB-231 and SW480 were also dependent, to a lesser extent, on HSP90β expression (Figure 1B and Supplementary Figure S1A and S1B). To induce resistance, cell lines were exposed to increasing concentrations of PU-H71, starting at 10 nM and scaling up gradually once the cells started to grow in the presence of the respective drug concentration, until a final PU-H71 concentration of 1 μM was reached. The time after which stable growth in the presence of 1 μM PU-H71 was achieved varied among the cell lines (A549 and SW480, approximately eight weeks; MDA-MB-231, approximately six months), pointing to the acquisition of different resistance mechanisms. Parental cell lines without PU-H71 (labeled “P” throughout the manuscript) were cultured in parallel during generation of the resistant cell lines (labeled “R” throughout the manuscript) to take into account possible effects of long-term culture that were unrelated to drug exposure. The differences in sensitivity to PU-H71 between the drug-sensitive parental cell lines and the resistant cell lines are displayed as half-maximal inhibitory concentration (IC_50_) values (Figure 1A) and IC_50_ curves (Figure 1C).

### Regained HSP90 function in PU-H71-resistant cell lines

**Figure 2:**
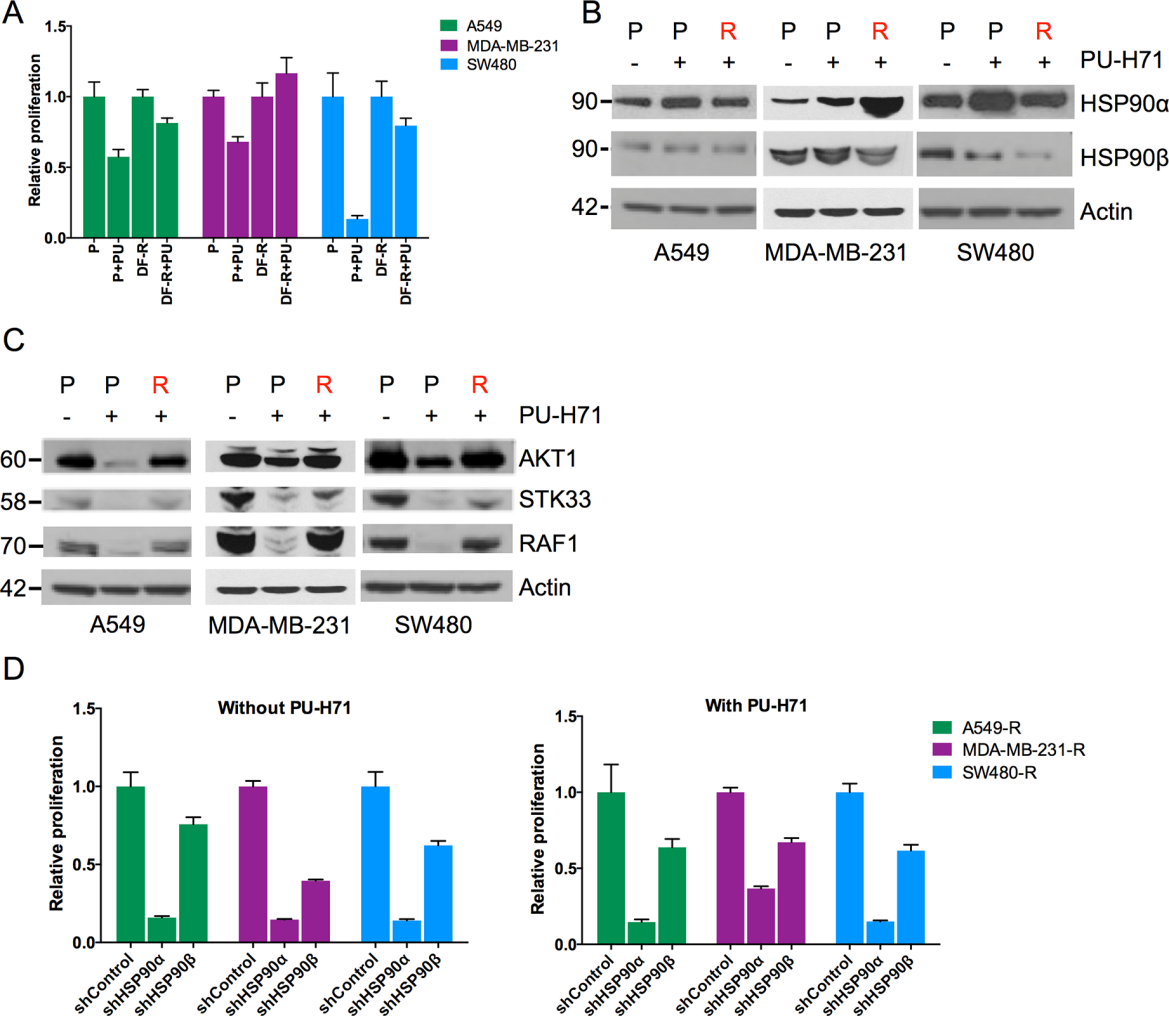
Regained HSP90 function in PU-H71-resistant cell lines (**A**) viability and proliferation of the indicated cell lines treated with 1 μM PU-H71 for four days. DF, drug-free (resistant cells grown six to eight weeks without PU-H71); PU, PU-H71. (**B** and **C**) western blot analysis of parental cell lines incubated with or without 1 μM PU-H71 for 24 hours, and resistant cell lines cultured continuously with 1 μM PU-H71. (**D**) viability and proliferation of PU-H71-resistant cell lines seven days after transduction with shRNAs targeting HSP90α or HSP90β, or a non-targeting control shRNA. Cells were either untreated (left panel) or incubated with 1 μM PU-H71 (right panel). Experiments were performed in triplicate, and one of two independent experiments is shown. Data are represented as mean ± SEM.

To begin to understand the mechanism(s) underlying the acquired insensitivity to PU-H71, we tested the stability of the resistance phenotype. A549-R, MDA-MB-231-R and SW480-R cells cultured without drug for a period of six to eight weeks maintained their viability and proliferation upon re-exposure to 1 μM PU-H71, pointing to an irreversible genetic alteration, as opposed to a transient mechanism such as epigenetic modifications or signaling pathway “rewiring”, underlying the resistance phenotype (Figure 2A).

To investigate whether changes of HSP90 itself might contribute to the acquisition of resistance, we first analyzed HSP90α and HSP90β protein levels in the parental cell lines, their resistant counterparts and parental cells treated with 1 μM PU-H71 for 24 hours. Acute HSP90 inhibition led to slightly increased HSP90α expression in all parental cell lines (Figure 2B). In addition, we observed substantially higher HSP90α levels in MDA-MB-231-R compared to MDA-MB-231-P cells, whereas HSP90 levels in the other resistant cell lines were either unchanged (HSP90α) or even slightly reduced (HSP90β) (Figure 2B). This finding suggested that in MDA-MB-231 cells, acquired resistance to PU-H71 could be attributed to upregulation of HSP90α.

To corroborate that resistance to PU-H71 was mediated, at least in part, through restoration of HSP90 function, we next assessed the chaperone activity of HSP90 by measuring the abundance of the client proteins AKT1, RAF1 and STK33, which have been linked to KRAS-mediated tumorigenesis [18]. As expected, all three clients were depleted upon acute PU-H71 treatment in the parental cell lines; in contrast, AKT1, RAF1 and STK33 protein levels in the resistant cell lines were comparable to those observed in untreated parental cell lines (Figure 2C). This result suggested that the resistant cells had regained HSP90 chaperone function in the presence of PU-H71. To verify this possibility, we determined via shRNA knockdown whether the resistant cells were still reliant on HSP90α and HSP90β. Similar to their parental counterparts (Figure 1B), the resistant cell lines showed reduced viability and proliferation upon knockdown of HSP90α or, to a lesser extent, HSP90β (Figure 2D, left panel, and Supplementary Figure S1A and S1C), an effect that was not enhanced further by the addition of PU-H71 (Figure 2D, right panel, and Supplementary Figure S1A and S1C). Together, these findings indicated that all three resistant cell lines had restored HSP90 function to maintain their viability and proliferation in the presence of PU-H71.

### Acquired PU-H71 resistance mediated by amplified HSP90α Y142N

**Figure 3:**
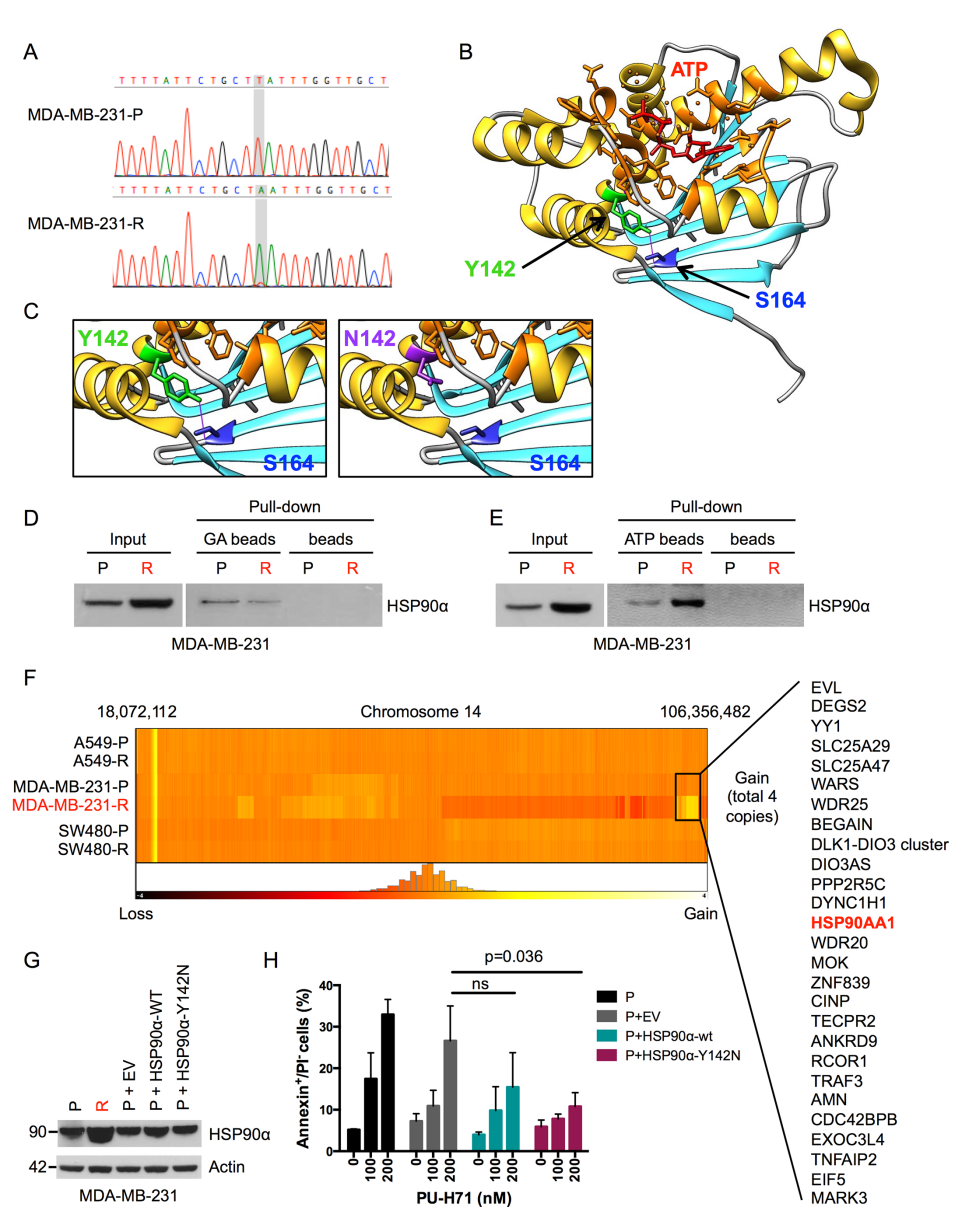
PU-H71 resistance mediated by mutation and amplification of *HSP90AA1* (**A**) portions of *HSP90AA1* exon 3 from parental and PU-H71-resistant MDA-MB-231 cells showing a c.T464A nucleotide change, resulting in a p.Y142N amino acid substitution, in the resistant cells (sequence numbering according to GenBank accession number AJ890083). (**B**) ribbon representation of the crystal structure of the HSP90α N-terminal domain bound to ATP (Protein Data Bank ID: 3T0Z) visualized using UCSF Chimera software (https://www.cgl.ucsf.edu/chimera/). The hydrogen bond between Y142 and S164 is indicated as purple line. Amino acids surrounding ATP in close proximity (< 5 angstrom) are indicated in orange. (**C**) enlarged structure of the protein region affected by the Y142N mutation. Left panel, wildtype HSP90α; right panel, HSP90α Y142N with disrupted hydrogen bond. (**D**) pull-down of HSP90α with geldanamycin (GA), showing reduced binding of GA to HSP90α Y142N in MDA-MB-231-R cells compared to wildtype HSP90α in MDA-MB-231-P cells despite higher expression of the mutant variant (input control). (**E**) pull-down of HSP90α with ATP, showing equal binding of ATP to HSP90α Y142N and wildtype HSP90α considering the different HSP90α expression levels (input control). (**F**) heatmap showing genomic copy numbers of part of chromosome 14 in all cell lines, demonstrating an amplified region containing *HSP90AA1* in MDA-MB-231-R. Other genes located in the amplified genomic region are indicated. (**G**) western blot analysis of MDA-MB-231-P, MDA-MB-231-R and MDA-MB-231-P cells stably transduced with an empty control vector (EV), wildtype (WT) HSP90α or HSP90α Y142N and cultured without PU-H71. (**H**) analysis of apoptosis 24 hours after incubation with PU-H71 in MDA-MB-231-P cells stably transduced with an empty control vector (EV), wildtype (WT) HSP90α or HSP90α Y142N. PI, propidium iodide. NS, not significant. Three independent experiments, mean ± SEM.

We next investigated whether the restoration of HSP90 function in PU-H71-resistant cells might be related to genetic alterations that render HSP90 insensitive to pharmacologic inhibition. Sequencing of all *HSP90AA1* and *HSP90AB1* coding exons in the parental and PU-H71-resistant cell lines revealed an *HSP90AA1* c.T464A mutation, resulting in a p.Y142N substitution in the N-terminal ATP-binding domain of HSP90α, in MDA-MB-231-R cells that was not present in the corresponding parental cell line (Figure 3A; Supplementary Figure S2A). No *HSP90AA1* or *HSP90AB1* coding region mutations were found in the other parental or resistant cell lines (data not shown).

In silico analysis using the SIFT (http://sift.jcvi.org) and PolyPhen (http://genetics.bwh.harvard.edu/pph2/index.shtml) algorithms predicted that the Y142N sequence variant affects the function of HSP90α (Supplementary Figure S2B). In addition, we assessed the potential functional consequences of the Y142N substitution based on the crystal structure of the HSP90α N-terminal domain (Protein Data Bank ID: 3T0Z). Residue Y142 forms a hydrogen bond with S164 that might be critical for maintaining the proper conformation of the adjacent ATP binding pocket (Figure 3B). Loss of this hydrogen bond as a result of the lower hydrophobicity and shorter side chain of asparagine in the Y142N variant might destabilize the conformation of the N-terminal domain and change the affinity of the ATP binding pocket for PU-H71 (Figure 3C). In accordance with this prediction, we found reduced binding of mutant HSP90α to the HSP90 inhibitor geldanamycin in MDA-MB-231-R cells despite higher expression of the mutant protein (Figure 3D). In contrast, HSP90α Y142N could bind ATP to a similar extent as wildtype HSP90α, indicating that the chaperone function of HSP90α Y142N remains intact (Figure 3E).

In addition to the HSP90α Y142N mutation, DNA copy number analysis using SNP microarrays identified a 3.5-Mb amplification on chromosome 14q32.31-q32.33 in MDA-MB-231-R cells, which resulted in two additional copies of *HSP90AA1* (Figure 3F). Given the markedly increased HSP90α protein levels in MDA-MB-231-R cells (Figure 2B), these findings suggested that MDA-MB-231-R cells had acquired resistance towards PU-H71 through mutation and amplification of the *HSP90AA1* locus, leading to overexpression of an HSP90α Y142N variant.

To test the ability of mutated HSP90α to confer PU-H71 resistance, we generated MDA-MB-231 cells stably transduced with wildtype HSP90α, HSP90α Y142N or an empty control vector (Figure 3G), followed by exposure to various concentrations of PU-H71 for 24 hours. Measurement of apoptosis by propidium iodide and annexin V staining and counting of live cells using trypan blue exclusion demonstrated that cell viability was rescued by HSP90α Y142N and, in part, wildtype HSP90α (Figure 3H, Supplementary Figure S3, data not shown). These findings support the notion that the HSP90α Y142N variant can confer resistance to HSP90 inhibition by PU-H71.

### Acquired PU-H71 resistance mediated by amplification and overexpression of ABCB1

**Figure 4:**
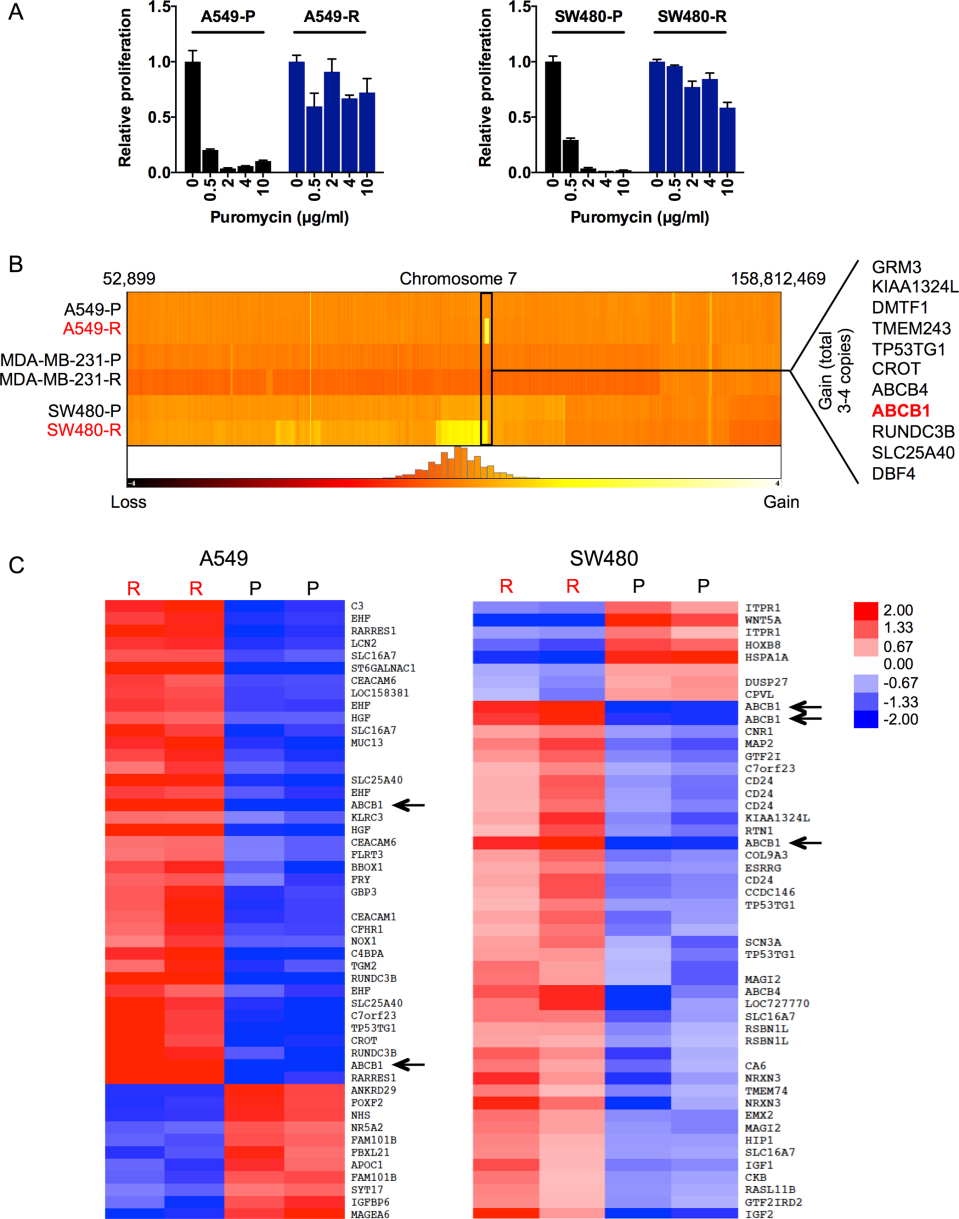
Amplification and overexpression of ABCB1 in A549-R and SW480-R cells (**A**) viability and proliferation of parental and resistant A549 and SW480 cells incubated with various puromycin concentrations for four days. (**B**) heatmap showing genomic copy numbers of part of chromosome 7 in all cell lines, demonstrating a commonly amplified region in A549-R and SW480-R cells containing the *ABCB1* gene. Other genes located in the commonly amplified genomic region are indicated. (**C**) heatmap showing the top 50 differentially expressed genes between resistant and parental A549 and SW480 cells. For each cell line, two different clones were analyzed.

Similar to MDA-MB-231-R, A549-R and SW480-R cells also showed stable PU-H71 resistance (Figure 2A) and had regained HSP90 chaperone function in the presence of PU-H71 (Figure 2C and 2D; Supplementary Figure S1). However, we detected no mutations or changes in gene copy number and/or mRNA expression of *HSP90AA1* and *HSP90AB1* in A549-R and SW480-R cells (Figures 2B and 3F, data not shown). When we exposed the PU-H71-resistant cell lines to puromycin for selection after transduction with lentiviral shRNA constructs (Figure 2D), we noticed that A549-R and SW480-R, but not their parental counterparts, were insensitive to puromycin. Incubation with various puromycin concentrations and measurement of viability and proliferation after three days demonstrated resistance of A549-R and SW480-R cells to puromycin concentrations as high as 10 μg/ml (Figure 4A).

Since PU-H71 and puromycin are both purine analogs, we hypothesized that A549-R and SW480-R cells might have acquired resistance to these compounds by upregulation of a transport protein that exports purine-like drugs. To identify such a transporter, we searched for DNA copy number changes using SNP microarrays and identified a 1.26-Mb gain on chromosome 7q21.12 that was common to A549-R and SW480-R (Figure 4B). This region contains the *ABCB1* gene encoding multi-drug resistance 1 (MDR1), a member of the superfamily of ATP-binding cassette transporters implicated in many cases of drug resistance [27, 28]. Consistent with the idea that *ABCB1* is a relevant target of the gained region on chromosome 7q21.12, gene expression array analysis demonstrated that *ABCB1* mRNA levels were strongly elevated in A549-R and SW480-R cells compared to their respective parental lines (Figure 4C).

**Figure 5:**
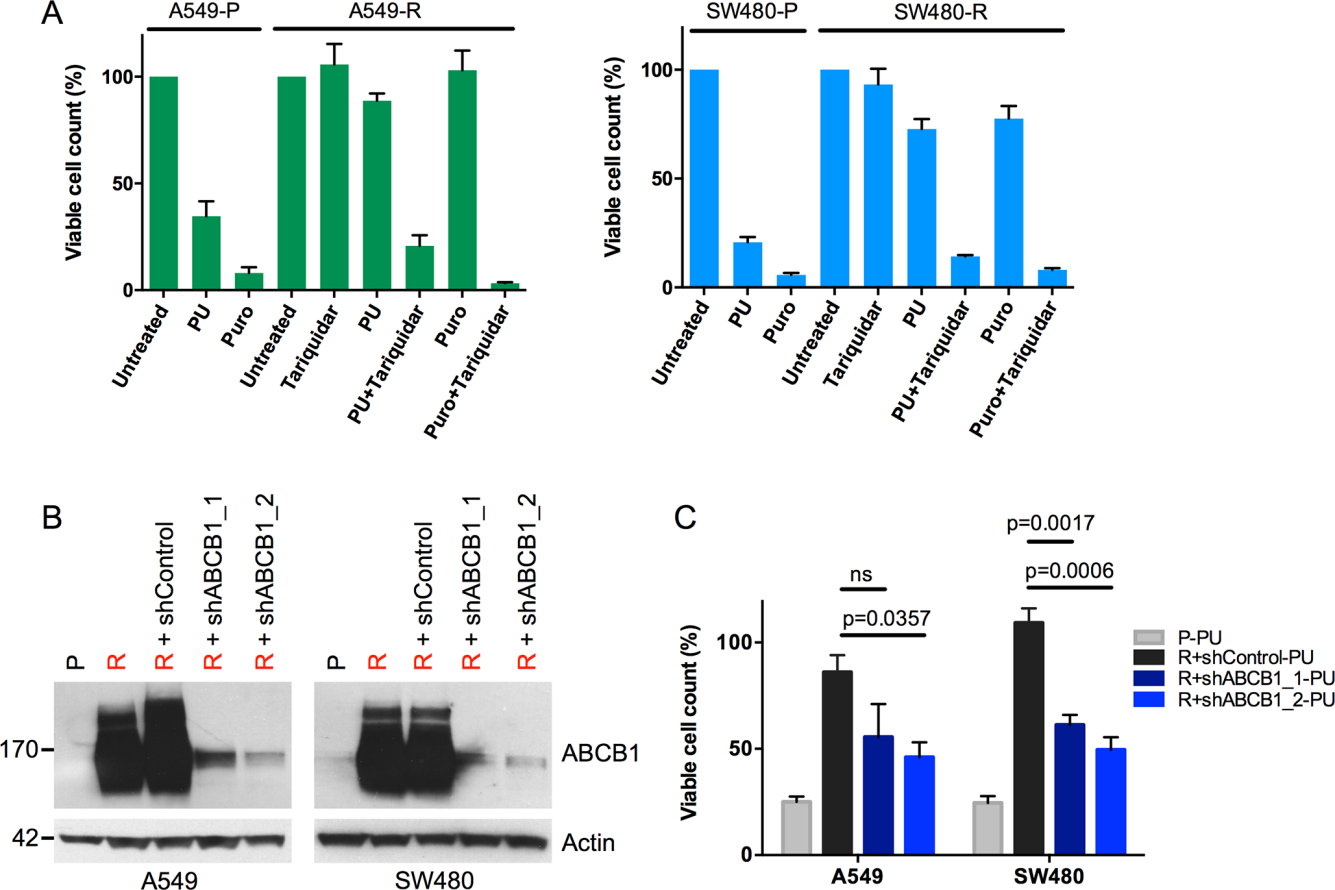
Reversal of PU-H71 resistance by MDR1 inhibition in A549-R and SW480-R cells (**A**) viability of parental and resistant A549 and SW480 cells treated with PU-H71 (PU), puromycin (Puro), tariquidar and combinations of tariquidar with PU-H71 and puromycin for four days. (**B**) Western blot analysis of parental and resistant A549 and SW480 cells seven days after transduction with shRNAs targeting ABCB1, or a non-targeting control shRNA. (**C**) viability of parental and resistant A549 and SW480 cells stably transduced with shRNAs targeting ABCB1, or a non-targeting control shRNA after treatment with 1 μM PU-H71 for four days. Relative cell counts are shown as percentages of untreated controls. Three to four independent experiments, mean ± SEM.

To evaluate whether the cross-resistance of A549-R and SW480-R cells could be attributed to enhanced drug export mediated by MDR1, we tested whether sensitivity to PU-H71 and puromycin was restored by treatment with tariquidar, a highly selective, non-competitive MDR1 inhibitor [29]. In support of this hypothesis, the combination of tariquidar with PU-H71 or puromycin decreased the viability of A549-R and SW480-R cells to a similar extent as PU-H71 or puromycin alone in drug-sensitive parental cells (Figure 5A). Furthermore, knockdown of ABCB1 using two different shRNA constructs also restored the sensitivity of A549-R and SW480-R cells towards PU-H71, as evidenced by reduced cell viability (Figure 5B and 5C). Taken together, these findings indicate that genomic amplification and overexpression of ABCB1 are the cause of PU-H71 resistance in A549-R and SW480-R cells.

### Sensitivity and resistance to structurally diverse HSP90 inhibitors

**Figure 6:**
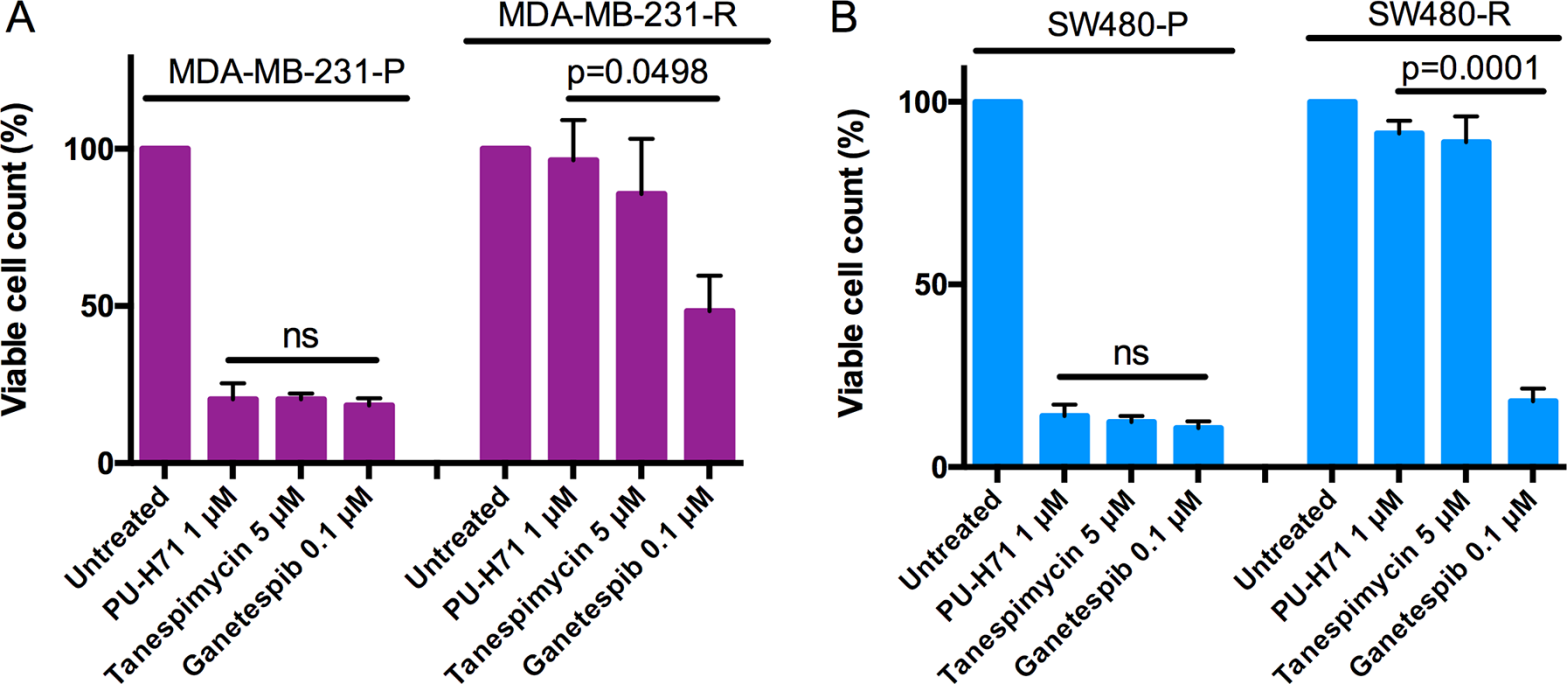
Sensitivity of resistant cells to structurally different HSP90 inhibitors (**A** and **B**) viability of parental and resistant MDA-MB-231 (A) and SW480 (B) cells treated with PU-H71, tanespimycin and ganetespib for four days. Three independent experiments, mean ± SEM.

We finally investigated whether resistance to PU-H71 could be overcome by treatment with two alternative, structurally distinct ATP-competitive HSP90 inhibitors that are currently in clinical development, the geldanamycin derivative tanespimycin (also known as 17-AAG) and the second-generation triazole ganetespib (also known as STA9090) [4, 30]. To this end, parental and PU-H71-resistant cell lines were incubated with 5 μM tanespimycin, 100 nM ganetespib and 1 μM PU-H71, and viable cells were counted after three days. As expected, parental MDA-MB-231 cells were highly sensitive to both tanespimycin and ganetespib. In contrast, PU-H71-insensitive MDA-MB-231-R cells showed complete cross-resistance to tanespimycin, consistent with the reduced binding capacity of geldanamycin to HSP90α Y142N (Figure 3D); however, ganetespib induced cell death in MDA-MB-231-R cells, albeit to a lesser extent than in the parental counterpart (Figure 6A). PU-H71-resistant SW480-R cells were also insensitive to tanespimycin, indicating export of this drug via MDR1, whereas ganetespib was able to induce cell death as effectively as in SW480-P cells (Figure 6B). Thus, HSP90-dependent cancer cells that have restored HSP90 function in the presence of PU-H71 via an HSP90*α* Y142N mutation or amplification and overexpression of ABCB1 may be targeted by alternative HSP90 inhibitors, such as ganetespib.

## DISCUSSION

Cancer cells are particularly dependent on the chaperone function of HSP90, which has prompted the development of several small-molecule HSP90 inhibitors that may provide a substantive therapeutic window. One reason for this dependence is that cancer cells harbor highly active, cancer cell-specific HSP90 that is present in multi-chaperone complexes, which have a markedly higher affinity towards HSP90 inhibitors than uncomplexed HSP90 in normal cells [31, 32]. In addition, cancer cells are frequently addicted to mutated or overexpressed oncoproteins whose proper folding and function is controlled and maintained by HSP90 [15, 33].

KRAS is a central node in signaling pathways that promote cell growth and survival, and is constitutively activated by mutations in one third of cancers. Previous attempts at direct targeting mutant RAS by disrupting its GTP-bound form or by interfering with its cell membrane localization have been unsuccessful [34], although compounds that irreversibly bind to the KRAS G12C protein, thereby changing its intrinsic preference for GTP over GDP, may provide a novel means to target this specific KRAS variant [35]. In addition, KRAS is not a HSP90 client and can therefore not be targeted directly by HSP90 inhibition [18]. This apparent “undruggability” has shifted the focus of anti-RAS strategies to the inhibition of more tractable downstream effectors such as the PI3K/AKT and RAF/MEK/ERK signaling pathways [36–39]. Certain KRAS effectors, such as AKT1 and RAF1, as well as STK33 that has been demonstrated to be preferentially required by mutant KRAS-dependent tumors, are kinases that are chaperoned by HSP90 [16, 18, 20]. Therefore, HSP90 inhibition might represent a viable therapeutic option for patients with KRAS mutant malignancies.

The promising *in vitro* and *in vivo* activity of HSP90 inhibitors in preclinical models of various cancer types has led to a large number of clinical trials testing the efficacy of these drugs as monotherapy and in combination with other targeted therapies in hematologic and solid-organ malignancies [5]; (https://clinicaltrials.gov). Based on the premise that, in analogy to other targeted cancer therapies, resistance to HSP90 inhibitors will inevitably occur, we sought to prospectively identify underlying mechanisms, which may guide sequential or simultaneous treatment strategies to overcome or prevent escape from HSP90 inhibition. By using KRAS mutant lung, breast and colon cancer cell lines that were rendered resistant to the purine analog PU-H71, we identified two genetic alterations that allowed adaptation to HSP90 inhibition.

First, MDA-MB-231 breast cancer cells restored HSP90 function in the presence of PU-H71 through an HSP90*α* Y142N mutation affecting the N-terminal ATP-binding pocket of HSP90α, which was accompanied by amplification and overexpression of the *HSP90AA1* locus. We hypothesize that the aberrant asparagine at position 142 disrupts the physiological interaction of Y142 and S164, thereby reducing the binding of PU-H71 to HSP90 without affecting the chaperone function and ATP hydrolysis. This prediction is supported by our observation that HSP90α Y142N can bind to ATP as efficiently as wildtype HSP90α, whereas the binding to geldanamycin is severely hampered. Along the same lines, the fungus *H. fuscoatra*, which produces the HSP90-inhibiting antibiotic radicicol, harbors a cytosolic HSP90 variant with mutation of a conserved amino acid in the N-terminal domain that confers resistance to radicicol without compromising ATP binding and hydrolysis [40]. Despite the same binding capacity of HSP90α Y142N and wildtype HSP90α to ATP, it is also plausible that HSP90α Y142N is slightly less efficient in ATP hydrolysis than the wildtype chaperone, but the high levels of HSP90α Y142N might compensate for its reduced functionality and allow MDA-MB-231-R cells to thrive in high PU-H71 concentrations. Importantly, the influence of HSP90*α* Y142N on the binding to HSP90 seems to vary among structurally different inhibitors, since MD-MB-231-R cells were cross-resistant to tanespimycin, whereas ganetespib was partially effective. In addition, mere overexpression of HSP90 might also induce resistance to HSP90 inhibition, as indicated by our finding that the viability of PU-H71-treated cells is partially rescued by exogenous HSP90α expression. It has been reported that elevated HSP90 levels buffer cancer cells from the increased proteotoxic stress associated with aneuploidy and are correlated with poor prognosis in various cancers [41, 42]. Thus, high intrinsic or acquired HSP90 expression could provide protection against HSP90 inhibitors independent of mutations that alter HSP90 structure and/or conformation.

Second, we identified amplification of the *ABCB1* gene and subsequent overexpression of the drug efflux pump MDR1 as the cause of acquired PU-H71 resistance in A549 lung cancer cells and SW480 colon cancer cells. The short time of about eight weeks that were necessary to obtain the resistant cell lines suggests that cells harboring a pre-existing *ABCB1* amplification were selected for by PU-H71 treatment, whereas the acquisition of a novel genetic alteration and subsequent clonal outgrowth of mutant cells would be expected to take longer, as observed with MDA-MB-231 cells that required six months to become resistant. This hypothesis is supported by the finding that cross-resistance to puromycin (another MDR1 substrate [43]) occurred again when we re-generated PU-H71-resistant A549 and SW480 cells (data not shown). Of note, overexpression of MDR1 has been shown previously to confer resistance to the structurally related inhibitors tanespimycin, geldanamycin and alvespimycin in cancer cell lines [44, 45]. Our data support these findings, but also indicate that ganetespib might not be substrate of MDR1.

MDR1 efficiently excludes many, structurally diverse compounds from cells, including various chemotherapeutic drugs, and MDR1 overexpression has been implicated in resistance to conventional cytotoxics as well as targeted agents [27, 28]. Also, a recent study showed that in nearly 8% of high-grade serous ovarian cancers with acquired resistance to paclitaxel, translocations and inversions of the 5′ regulatory region of the *ABCB1* gene lead to MDR1 overexpression compared to patient-matched, paclitaxel-sensitive primary tumors [46]. Given that the majority of ongoing clinical trials with HSP90 inhibitors are conducted in patients with advanced malignancies, they might underestimate the efficacy of these drugs due to frequent MDR1 overexpression in cancers that have failed previous treatment. Furthermore, measurement of MDR1 expression before and during HSP90 inhibitor administration may enable enrichment of trial cohorts for patients more likely to respond, and guide development of rational combination therapies. For example, MDR1-overexpressing SW480-R cells show resistance to both PU-H71 and tanespimycin but are sensitive to ganetespib, illustrating that knowledge of MDR1 expression may aid in selecting a specific HSP90 inhibitor. Finally, despite the mixed outcomes of phase 3 clinical trials with MDR1 inhibitors, mostly due to high toxicity in combination with cytotoxic agents, co-administration of PU-H71 and new-generation MDR1 inhibitors may be feasible given the high affinity of PU-H71 for active HSP90 [31, 32] and its specific targeting of complexes comprised of HSP90 and oncogenic fusion proteins in certain cancers [47].

Taken together, our data identify potential mechanisms of resistance to pharmacologic HSP90 blockade that can aid in patient selection and guide the development of additional HSP90 inhibitors or rational combination therapies.

## MATERIALS AND METHODS

### Cell lines and inhibitors

All cell lines were obtained from ATCC and maintained under standard conditions. Cell line identity and purity were verified using the Multiplex Cell Authentication and Contamination Tests (Multiplexion). PU-H71 was generated and provided by the laboratory of G. Chiosis as previously described [47]. Puromycin was obtained from Sigma-Aldrich. Ganetespib, tanespimycin and tariquidar were purchased from Selleck. From each PU-H71-resistant cell line, three to five clones were generated by seeding single cells into 96-well plates.

### Constructs and lentiviral transduction

The *HSP90AA1* cDNA was obtained from Open Biosystems. To generate the p.Y142N mutant, a c.T464A mutation was introduced in exon 3 of *HSP90AA1* (NCBI accession AJ890083 and NP_005339.3) using the QuikChange XL Site-Directed Mutagenesis Kit (Stratagene), and the presence of the mutation was confirmed by Sanger sequencing. The wildtype and mutant *HSP90AA1* open reading frames were cloned into the pLenti6.2/V5-DEST lentiviral expression vector using Gateway Technology (Invitrogen). RNA interference experiments were performed using pLKO.1 lentiviral short hairpin RNA (shRNA) vectors from the TRC-Hs 1.0 (Human) shRNA library: shHSP90AA1_1, TRCN0000001025; shHSP90AB1_1, TRCN0000008748; shABCB1_1, TRCN0000059685; shABCB1_2, TRCN0000059687. Generation of viral supernatants and viral transduction were performed as previously described [16].

### Western blot and pull-down analyses

Whole-cell protein lysates were generated and analyzed by western blotting as described before [18]. Whole-cell protein extracts for HSP90 pull-down with geldanamycin were prepared with lysis buffer containing 50 mM HEPES, 150 mM NaCl, 1% Triton-X100, protease and phosphatase inhibitors. Lysates (250 μg) were incubated with 40 μg geldanamycin-biotin (Sigma-Aldrich) and 125 μl settled NeutrAvidin Agarose Resin (Thermo Scientific) at 4°C. Immobilized proteins were washed three times, resuspended in Laemmli buffer and subjected to SDS-PAGE and western blotting. The HSP90 pull-down with ATP beads was performed using the Pierce Kinase Enrichment Kit with ATP Probe (Thermo Scientific) according to the manufacturer's instructions. The following antibodies were used: anti-β-actin (Sigma-Aldrich), anti-RAF1 (#9422, Cell Signaling), anti-AKT1 (2H10, Cell Signaling), anti-STK33 (4F7, Abnova), anti-HSP90β (D-19, Santa Cruz), anti-HSP90α (AB3466, Millipore) and anti-p-glycoprotein (anti-ABCB1) (C219, Millipore).

### Sequencing of *HSP90AA1* and *HSP90AB1*

Genomic DNA was isolated from PU-H71-sensitive or resistant cell lines using the PureLink Genomic DNA Mini Kit (Invitrogen). *HSP90AA1* and *HSP90AB1* coding exons were amplified using previously described primers [48] and standard PCR conditions with adjustments for annealing temperature and extension time, and amplified fragments were subjected to direct Sanger sequencing. Chromatogram sequence alignment of *HSP90AA1* was performed using MacVector 13.5.5.

### Cell viability and proliferation assays

To evaluate the effects of drug treatment or shRNA knockdown, 2,000 cells per well were plated in 96-well plates with indicated drug concentrations or five days post lentiviral transduction, respectively, and cell viability and proliferation were analyzed after two to four days using the CellTiter 96AQ_ueous_ One Solution Proliferation Assay (Promega). Cell viability was also assessed by trypan blue exclusion using a Vi-CELL Cell Viability Analyzer (Beckman Coulter). For this assay, 100,000 cells per well were plated in 24-well plates, drugs were added the next day, and suspension and adherent cells were analyzed four days post drug treatment.

### Gene expression profiling and single-nucleotide polymorphism array analysis

Gene expression profiling and single-nucleotide polymorphism (SNP) array analysis were performed as previously reported [49]. In brief, total RNA was isolated from PU-H71-sensitive and resistant clones using the RNeasy Mini Kit (Qiagen), and gene expression was profiled using GeneChip Human Genome U133 Plus 2.0 Arrays (Affymetrix) according to the manufacturer's recommendations. The complete microarray dataset is available at the Gene Expression Omnibus (http://www.ncbi.nlm.nih.gov/projects/geo; accession number GSE85734). Genomic DNA was isolated from bulk PU-H71-sensitive and resistant lines using the PureLink Genomic DNA Mini Kit (Invitrogen), and genomic profiling was performed with Genome-Wide Human SNP Arrays 6.0 (Affymetrix) according to the manufacturer's recommendations. After generation of raw data (CEL files) using the Genotyping Command Console version 2.0 (Affymetrix), paired analysis of PU-H71-sensitive and resistant samples was performed as previously reported [50]. The complete SNP array dataset is available at the Gene Expression Omnibus (accession number GSE85734).

### Apoptosis analysis and flow cytometry

Annexin V and propidium iodide staining were performed using the Annexin V-PE Apoptosis Detection Kit (eBiosciences) per manufacturer's protocol. Flow cytometric analysis was performed on a FACSCalibur instrument (Becton Dickinson), and analysis was performed using FlowJo software.

### Statistical analysis

Statistical analysis was performed using an unpaired *t*-test unless otherwise specified. Computations were performed using GraphPad Prism 6.

## SUPPLEMENTARY MATERIALS FIGURES


