# Supplementary material for: Prospective identification of resistance mechanisms to HSP90 inhibition in KRAS mutant cancer cells
Source: Oncotarget. 2016 Dec 9;8(5):7678–90. doi: 10.18632/oncotarget.13841 (PMC5352352; doi:10.18632/oncotarget.13841)
Supplement: Supplementary file 1 [file oncotarget-08-7678-s001.pdf]

Prospective identification of resistance mechanisms to HSP90 inhibition in KRAS mutant cancer cells

Supplementary Materials

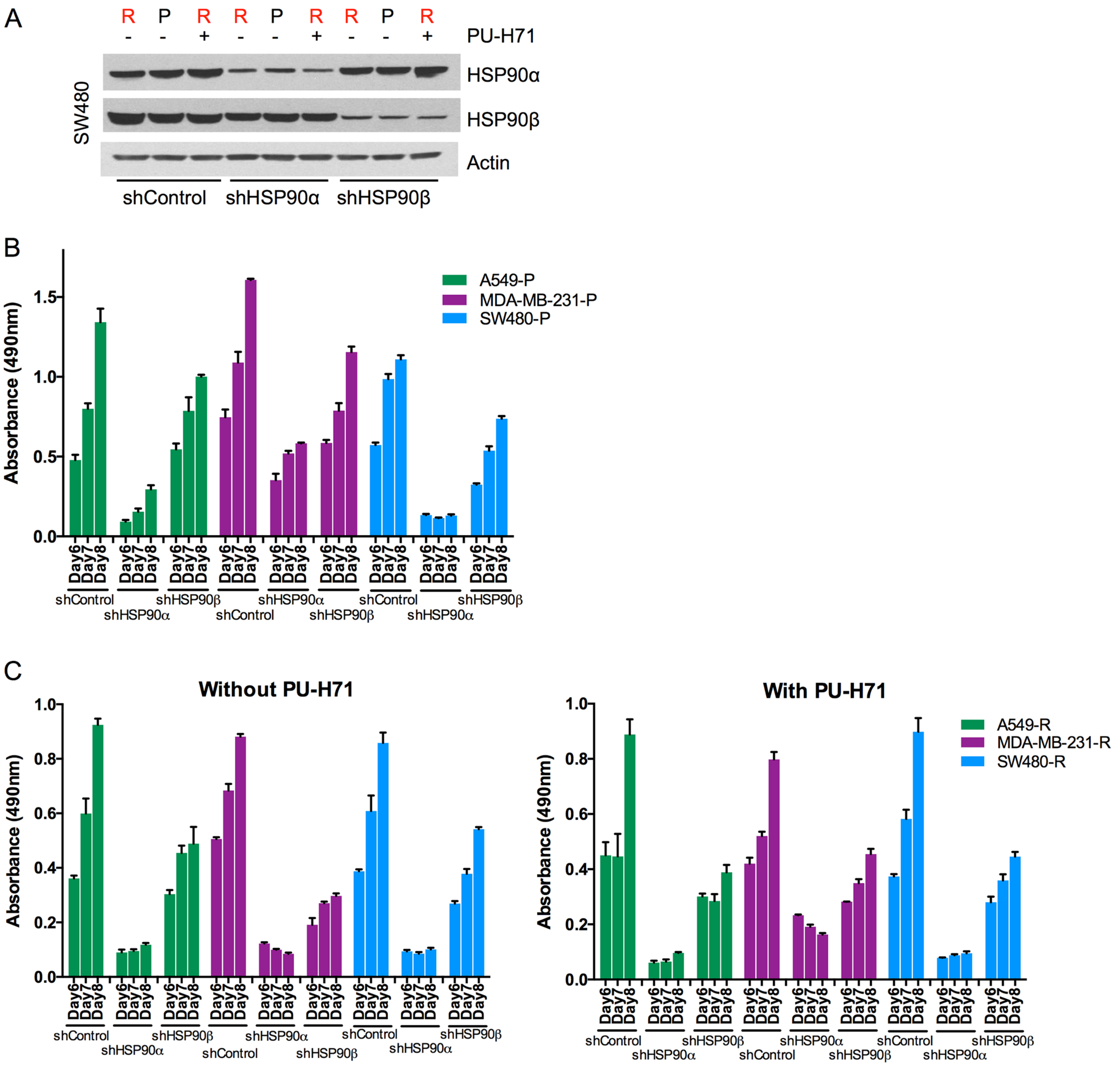

Supplementary Figure S1: Dependence of parental and PU-H71-resistant cell lines on HSP90α and HSP90β. (**A**) Representative western blot of parental and resistant SW480 cells cultured with or without PU-H71 and transduced with a non-targeting control shRNA and shRNAs targeting HSP90α and HSP90β. (**B**) Viability and proliferation of parental cell lines six to eight days after transduction (two to four days post drug treatment) with a non-targeting control shRNA and shRNAs targeting HSP90α and HSP90β. (**C**) Viability and proliferation of PU-H71-resistant cell lines six to eight days after transduction with a non-targeting control shRNA and shRNAs targeting HSP90α and HSP90β treated with (right panel) or without (left panel) 1 μM PU-H71.

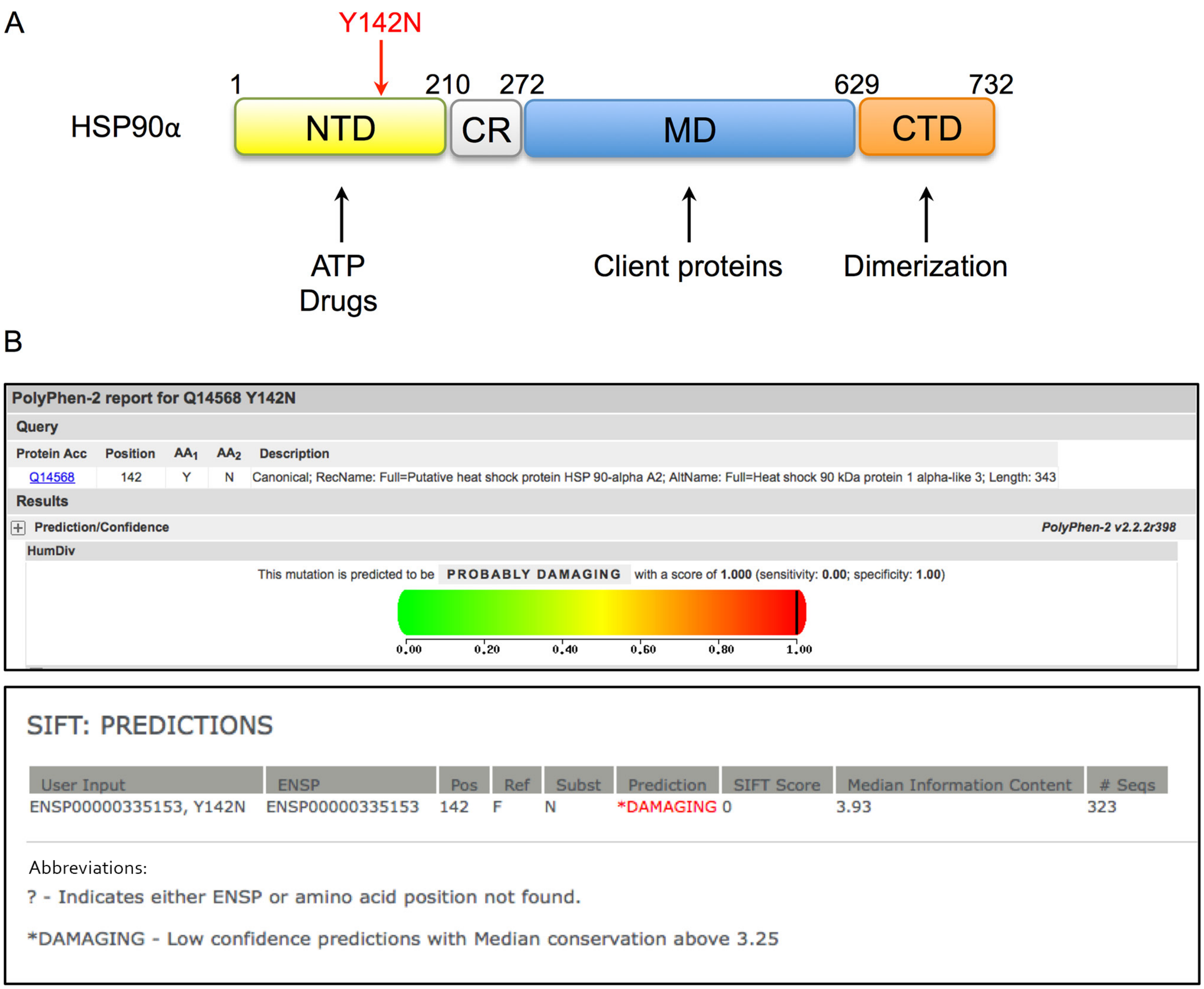

Supplementary Figure S2: ***In silico*** **prediction of the functional consequences of the Y142N mutation in HSP90α.** (**A**) Schematic representation of HSP90α isoform 2. Domains, their binding properties, and the localization of the Y142N mutation are indicated. NTD, N-terminal domain; CR, charged region; MD, middle domain; CTD, C-terminal domain. (**B**) Prediction of the potential effect of the Y142N mutation on HSP90α protein function using PolyPhen-2 (upper panel) and SIFT (lower panel) software tools.

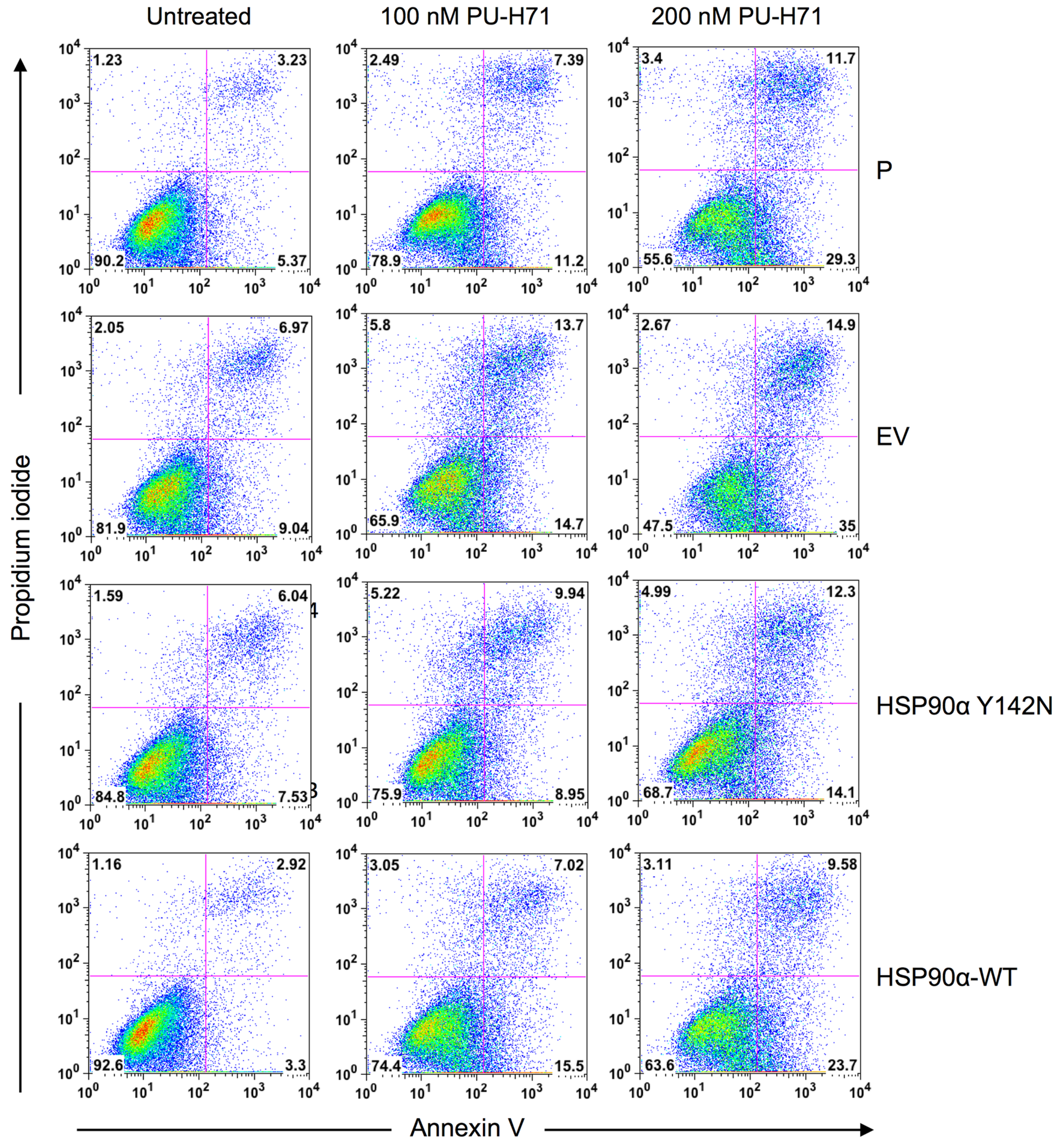

Supplementary Figure S3: Rescue of cell viability in MDA-MB-231 parental cells by HSP90α Y142N. Annexin V/propidium iodide staining of MDA-MB-231 cells stably transduced with an empty control vector (EV), HSP90α Y142N and wildtype (WT) HSP90α and treated with or without PU-H71 for 24 hours. P, parental. Numbers indicate percentages of cells.
